# Co-application of selenium nanoparticles and *Aloe vera* gel alleviates chromium toxicity in spinach by modulating antioxidative defense mechanisms

**DOI:** 10.3389/fpls.2026.1786570

**Published:** 2026-04-29

**Authors:** Sahar Sajjad, Arslan Khan, Azeem Mushtaq, Mason T. MacDonald, Muhammad Hamza, Saba Rasheed, Qamar uz Zaman, Mona S. Alwahibi, Shikha Alnamer, Afzal Hussain

**Affiliations:** 1 Department of Environmental Sciences, The University of Lahore, Lahore, Pakistan; 2 Department of Plant, Food and Environmental Sciences, Faculty of Agriculture, Dalhousie University, Bible Hill, NS, Canada; 3 Department of Botany and Microbiology, College of Science, King Saud University, Riyadh, Saudi Arabia

**Keywords:** *Aloe vera* gel, antioxidants, chromium, nanoparticles, oxidative stress, priming

## Abstract

Soil contamination by chromium (Cr) has severely degraded soil the health, which poses a serious threat to sustainable agricultural production worldwide. This present study investigated the combined application of *Aloe vera* gel and selenium nanoparticles (Se-NPs) to enhance the phytoremediation potential of spinach grown in a Cr-contaminated soil. It was hypothesized that the combined use of Se-NPs and *Aloe vera* gel would reduce Cr toxicity in spinach via improving the antioxidant defense system. A pot experiment was conducted to assess the role of *Aloe vera* gel seed priming and foliar applied selenium (Se) to alleviate the Cr-induced stress in spinach plants. The current experiment followed a completely randomized design (CRD) and consisted of two factors: factor A included treatments with and without *Aloe vera* gel as its seed priming application, and factor B included foliar application of the Se-NPs at different levels (0, 0.5, 1.0, and 2 mg/L). Each experimental treatment was replicated three times. A two-way analysis of variance (ANOVA) was performed using Statistix 8.1, while correlation analysis, heat map visualization and principal component analysis (PCA) was performed using R-Software. The Se-NPs and *Aloe vera* gel significantly increased biomass by up to 48%, chlorophyll a by 37%, soluble protein by 99% and reduced malondialdehyde and electrolyte leakage (EL) by over 40% at 2 mg L^-^¹ Se NPs (P ≤ 0.05). Combined *Aloe vera* and Se NPs also restricted the Cr accumulation in leafy parts of spinach plants. Cr inhibited spinach growth and reduced photosynthesis by inducing over-production of the ROS and negatively affected the antioxidant defense system. However, the combined application of Se-NPs and *Aloe vera* gel significantly enhanced spinach growth and photosynthesis, alleviating Cr-induced stress through scavenging ROS by strengthening the plant anti-oxidative defense system, while Cr level remained under the threshold level (1.30 µg/g). The use of NPs represents an eco-friendly and cost-effective approach, which may serve as a green alternative to conventional methods used for heavy metal remediation. Mechanistically, these improvements were linked to up-regulation of the antioxidant enzymes and reduced accumulation of the ROS, suggesting enhanced redox homeostasis under stressful conditions. Our results demonstrate that the combined treatment of Se-NPs and *Aloe vera* gel significantly enhances spinach tolerance to chromium toxicity and is a good approach to promote phytoremediation and crop production in contaminated soils.

## 1 Introduction

Heavy metal (HM) contamination is a serious environmental challenge that reduces crops yield, degrades food quality, and poses severe humans health concerns (Angon et al., 2024). Heavy metal pollution arises from both natural processes (e.g. weathering of rocks, volcanic eruptions) and anthropogenic activities (e.g. mining, industrialization, agrochemical use, and urbanization) which disturbs the natural bio-geochemical cycles (Jadaa and Mohammed, 2023). Environmental contamination has therefore become a global concern, as pollutants from the industrial and human activities move into soil, crops, animals, and ultimately humans via the food chain (Noor et al., 2023).

HM contamination is a significant ecological and agronomic threat due to its persistence, bioaccumulation and trophic transfer. Toxic metals including chromium (Cr), cadmium (Cd), lead (Pb) and mercury (Hg) are accumulated in soils and aqueous systems; so reduce plants physiological processes and crops productivity (Angon et al., 2024). In plants, growth is affected by heavy metals due to disturbances in photosynthesis, nutrients uptake, and redox equilibrium, which leads to growth inhibition, chlorosis, necrosis and plant death in severe cases (Riyazuddin et al., 2021). Among these contaminants, Cr is very critical, due to its widespread industrial use, high mobility in soil-plant matrices, and persistence in the environment over time. Cr represents a critical challenge both for the sustainable production of agricultural activities as well as for the integrity of the ecosystem, especially in areas with intensive industrial activities.

Cr is a toxic HM widely used in the production of stainless steel, textile dyeing and electroplating and leather industry (Komal et al., 2024). In Pakistan, the primary source of Cr pollution is leather processing units, with more than 600 tanneries located in three major cities of Pakistan, such as Karachi, Sialkot, and Kasur. HM stress from tannery wastewater increases Cr uptake in rice and reduces crops yield (Zaman et al., 2021). Human exposure to such contaminants occurs mainly through dietary intake (Ali et al., 2018). Therefore, sustainable mitigation strategies are much needed in order to reduce the adverse effects of Cr on plants and human health.

Sustainable approaches to reduce HM toxicity in plants include phytoremediation, the use of organic amendments such as biochar, compost, manure, and the application of biostimulant-like nanoparticles (NPs) or *Aloe vera* gel that improve plant tolerance to metal stress (Abideen et al., 2022). Previously has been reported that co-composed biochar and titanium dioxide NPs Enhanced plant growth and enhance Cd uptake in wheat (Chen et al., 2023). In addition, agronomic practices such as crop rotation, use of tolerant varieties, and improved soil management also reduce metal uptake in edible plant parts. These strategies not only improve food safety and soil health but also support global sustainability goals via protecting ecosystems and ensuring safe crops production. Furthermore, the combined application of zinc oxide and iron NPs have been shown to improve growth and antioxidants activity in Red Sails lettuce while reducing Cr uptake in contaminated soil (Sameer et al., 2024).

*Aloe vera* gel, with its antioxidant, anti-stress, and growth-promoting properties, can increase spinach tolerance against abiotic stresses including drought and salinity. Meanwhile, Se-NPs enhance nutrients uptake, immune responses, and resistance to oxidative stress. Selenium protects plants from toxic trace elements by promoting growth and photosynthesis, alleviating oxidative damage via effective control of ROS (Rizwan et al., 2021).

The eco-friendly alternatives reduce dependence on synthetic chemicals, enhance crop yields, and improve nutritional quality, thereby supporting sustainable farming while contributing to environmental protection and food security. A recent study demonstrated the positive effects of co-composted biochar and titanium dioxide NPs on Cd accumulation and wheat growth (Chen et al., 2023). Likewise, SiO_2_-NPs have been reported to enhance the growth of *Brassica napus* L. and strengthen its antioxidative defense system by reducing Cr uptake (Huang et al., 2024). Further studies show that biochar and ZnO NPs are emerging as sustainable tools to mitigate both biotic and abiotic stresses in plants (Ahmad et al., 2024).

The current study focuses on a sustainable strategy to mitigate Cr-induced stress in *Spinacia oleracea*, a globally consumed leafy vegetable known for its vitamins, minerals and antioxidants properties (Murcia et al., 2020). The Se-NPs primarily enhance antioxidants enzymatic defense system and regulate redox homeostasis, while *Aloe vera* gel delivers bioactive compounds that contribute to membrane stabilization, osmoprotection, and ROS scavenging. Se-NPs have shown potential to reduce oxidative damage and improve plants growth under HMs stress (Shang et al., 2023). However, the combined use of Se-NPs with *Aloe vera* gel for Cr detoxification is mainly unexplored. The synergistic effect of combining nano-based antioxidants with plant-derived bioactive compounds remain little explored.

We hypothesized that the individual and synergistic application of Se-NPs and *Aloe vera* gel can mitigate Cr-induced oxidative stress in spinach via antioxidant defense enhancement, cellular membrane stabilization, of ROS reduction and subsequent improvements in growth, yield and phytoremediation efficiency. Thus, the objectives of the present study were: (i) to evaluate the effects of Se-NPs and *Aloe vera* gel, alone and in combination, on the agronomic and physiological characteristics of spinach under Cr stress; (ii) to assess their effects on the antioxidant enzyme activities and oxidative stress markers; and (iii) to determine their role in Cr uptake and their phytoremediation potential.

## 2 Materials and methods

### 2.1 Experimental site, design and treatments

A pot experiment was conducted to assess the individual and synergistic effect of *Aloe vera* gel and foliar-applied Se-NPs in mitigating chromium toxicity in spinach. A completely randomized design (CRD) with a 2×4 factorial arrangement was used and conducted in triplicate.

Spinach plants were grown in a Cr-contaminated soil under two experimental factors. Factor A comprised of seed priming treatments with and without *Aloe vera* gel, while the Factor B involved foliar application of Se-NPs at 4 concentrations: 0, 0.5, 1.0, and 2 mg/L.

### 2.2 Preparation of *Aloe vera* gel

Fresh leaves of *Aloe vera* were cut and washed to remove dirt from the surface of leaves. Leaves were cut with a knife to collect the internal gel of *Aloe vera* in a beaker, then mixed in a blender to get a homogenous mixture. To reduce microbial contamination while preserving bioactive compounds, the blended gel was gently heated at 80 °C for 10 minutes then allowed to cool at room temperature. This mild heating ensured partial sterilization without significant degradation of phenolics, polysaccharides, and other bioactive compounds. The prepared gel was analyzed for pH, total soluble solids, and antioxidant content before application.

### 2.3 Plant material and growth conditions

Random soil samples were collected from the study site at a depth of 0–30 cm, air-dried, and was sieved through a 2 mm mesh. *Aloe vera* gel (10% w/w) was prepared separately and stored in plastic bottles for application. Spinach seeds were soaked in a diluted *Aloe vera* gel solution for 2–3 days until sprouting. The pot experiment was conducted using three replicates per treatment, with five seedlings per pot, and each pot serving as an independent experimental unit for statistical analysis.

For the green synthesis of Se-NPs the turmeric (*Curcuma longa*) root extract was obtained by mixing in 80:20 (ethanol: acetone) and was mixed with sodium selenite solution using magnetic stirring at 50-60 °C. The formed precipitates were filtered by Whatman filter paper No. 1 and then dried in an oven at 50 °C to obtain green synthesized Se-NPs. The biosynthesized Se-NPs showed a crystalline structure with an average particle size of 32 nm determined by X-ray diffraction (XRD) analysis. Scanning electron microscopy (SEM) exhibited that their morphology was hexagonal. Fourier-transform infrared (FTIR) spectroscopy was used to confirm the presence of stabilizing functional groups of the turmeric extract, i.e., hydroxyl (O-H), aliphatic (C-H), alkene (C=C), and ether (C-O-C) functional groups. Meanwhile, ultraviolet-visible (UV-Vis) spectroscopy showed a strong maximum absorbance at 370 nm, which supported the formation of the required NPs.

Spinach seeds were soaked in selected concentrations of diluted *Aloe vera* gel for 2–3 days in petri plates, while untreated seeds served as control. The plates were kept moist until germination started, and five uniform seedlings were kept in each plate. After germination, seedlings were transferred to plastic pots containing Cr-contaminated soil. Tap water was used for irrigation when needed, and routine care was provided throughout the experiment. The NPK fertilizer (120:50:25) was applied, and Se-NPs were applied as foliar spray (500 mL) with one week interval in four applications (Pietrzykowski and Krzaklewski, 2007; Zohaib et al., 2023).

### 2.4 Data collection methods

#### 2.4.1 Growth parameters

Spinach plants were harvested and separated into roots and shoots for the measurement of growth and biomass. Plants height, leaf length, width, and leaf area were measured, and total the number of leaves per plant was counted. Fresh weights of roots and shoots were determined after washing with distilled water, while dry weights were measured after oven-drying the samples at 70 °C for two days.

#### 2.4.2 Photosynthetic parameter

Chlorophyll content was measured by following the procedure given by Lichtenthaler (1987). Photosynthetic attributes were measured from the uppermost green leaves using a portable analyzer of infrared gases (ADC Bioscientific, Hoddesdon, UK) after 35 days of germination. The measurements were taken between 9:00 to 12:00, when light intensity was maximum (Emanuil et al., 2020).

#### 2.4.3 Biochemical parameters

Fresh leaf tissue (0.2 g) was cut into pieces and put in test tubes with 10 mL of distilled water. The tubes were incubated in a water bath at 32 °C for 2 hours, followed by Dionisio-Sese and Tobita (1998). Afterwards, the tubes were cooled, and the electrical conductivity (EC1) of the supernatant was recorded. Then the samples were autoclaved for 20 min at 121 °C. After cooling at 25 °C, electrical conductivity (EC2) was recorded, where electrolyte leakage (EL) was calculated with the formula: EL = EC1/EC2 × 100.

MDA contents were determined using a thiobarbituric acid (TBA) assay. Fresh leaf tissues (0.1 g) were homogenized in 1 mL of 0.1% TCA and centrifuged for 14 min at 12,000 g. Then supernatant (1 mL) was mixed into 1 mL of 20% TCA containing 0.5% TBA, heated at 92 °C for 28 min, and centrifuged at 7,500 g for 5 min. Afterwards, the absorbance was measured at 532 and 600 nm, and MDA contents were expressed as nmol/g FW using 5% TCA as blank (Grintzalis et al., 2013). MDA concentration in leaves (0.5 g) was measured using the TCA method, and absorbance was recorded at 532 and 600 nm using spectrophotometer.

The H_2_O_2_ contents were recorded in leaf samples 0.25 g, which were homogenized in 5 mL TCA. After centrifugation (12,000 rpm, 15 min) and cooling, 0.5 mL of supernatant was mixed into 0.5 mL of phosphate buffer (pH 7) and 1 mL KCl (1 M). Then, absorbance was recorded at 390 nm using a spectrophotometer (Eimar et al., 2012).

#### 2.4.4 Enzymatic antioxidant activities

Samples of freshly expanded spinach leaves (1.0 g) were immediately frozen by immersion in liquid nitrogen and ground into a fine powder using a mortar and pestle. The grinded material was homogenized in 0.05 M phosphate buffer (pH 7.8) and filtered using 10 successive layers of muslin cloth. The homogenized mixture was centrifuged at 12000 × g for 10 min at 4 °C. The supernatant was collected and used for all further enzymatic assays.

Peroxidase (POD) and superoxide dismutase (SOD) activities were measured followed by Velikova et al. (2000) and Zhang et al. (1992), while ascorbate peroxidase (APX) activity was recorded followed by Nakano and Asada (1981). Catalase (CAT) activities were recorded according to the procedure given by Aebi (1974), using 300 µL of protein extract in 2.8 mL of phosphate buffer (50 mM, pH 7.0) supplemented with 2 mM of EDTA and 300 mM of H_2_O_2_. Catalase activity was calculated from the decrease in absorbance at 240 nm using an extinction coefficient of 39.4 mM^-1^ cm^-1^. The concentration of soluble protein was measured followed by Bradford (1976) method using bovine serum albumin (mg mL^-1^) as the standard solution and Coomassie Brilliant Blue G-250 as the chromogenic reagent. All the enzymatic activities were recorded using a calibrated spectrophotometer and each assay was performed in triplicate (n = 3) to ensure reproducibility.

#### 2.4.5 Analysis of soluble proteins, soluble sugars, amino acids and proline

Soluble protein contents in spinach leaves were measured using the Coomassie brilliant blue G-250 method. Leaf samples (0.1 g) was homogenized in mortar by using 1 mL phosphate buffer solution (PBS, pH 7.8) and a 10 mL centrifuge tube was filled with the resultant grinding fluid. Two milliliters of PBS were used to wash the mortar twice to remove as much of the grinding fluid as possible. These washing solutions were then placed into the same centrifuge tube. Following that, the tube was centrifuged at 10,000 rpm for 15 min at 4 °C. After centrifugation, the supernatant was transferred to a new centrifuge tube, which was subsequently stored in an icebox to preserve a low temperature. Then 0.1 mL of the supernatant from the new tube was transferred to another centrifuge tube, 5 mL of Coomassie brilliant blue G-250 were added to this tube, and everything was well combined.

The solution was mixed thoroughly for 2 min, transferred to a cuvette and subjected to colorimetric measurement at 595 nm with a spectrophotometer. Soluble sugars contents were recorded from fully expanded leaves at 50% of the flowering stage under different treatment conditions using the enthrone method, with 0.1 mg mL^-1^ glucose in distilled water as a standard followed by Dubois et al. (1956). Absorbance were measured at 620 nm, and sugar contents were reported as milligrams of glucose per gram of fresh weight (mg glucose g^-1^ FW).

Total free amino acids (AA) were determined in leaf samples collected at 50% of flowering stage under different treatment conditions by using Ninhydrin reagent, with absorbance readings recorded at 570 nm following the method given by Yemm et al. (1955). Proline content was measured according to the method described by Bates et al. (1973). Fresh plant tissue (0.5 g) was homogenized in 10 mL of 3% sulfosalicylic acid and filtered. The filtrate (2 mL) was mixed with 2 mL of acid ninhydrin, and glacial acetic acid (2 mL) in test tube and then heated for 1 h at 100 °C in the water bath, then cooled and add 4 mL of toluene. The contents were mixed thoroughly. A spectrophotometer was used to calculate absorbance at 520 nm.

### 2.5 Chromium accumulation and translocation factor

Spinach shoots samples were digested by using a diacid mixture of nitric acid and perchloric acid (HNO_3_: HClO_4_ vs. 4:1). To check the Cr uptake in root and shoot samples, an atomic absorption spectrophotometer (Perkin-Elmer, Model 3300) was used. The root-to-shoot translocation factor (TF) was calculated to quantify the internal mobility of chromium in the plant. The TF was measured using the following formula: TF = Cr concentration in shoot/Cr concentration in root.

### 2.6 Statistical analysis

Statistical analyses were performed using Statistix 8.1. Data were examined for normality and homogeneity of variance through inspection of residual plots prior to ANOVA. A two-way factorial ANOVA was used to measure differences between treatments. Significant difference was detected at (P ≤ 0.05), *post-hoc* Tukey’s HSD test was applied for pairwise comparisons. All results are presented as mean ± standard deviation (SD), and P-values (P ≤ 0.05) were considered statistically significant. Correlation analysis, heat map visualization and principal component analysis (PCA) was completed using R-Software.

## 3 Results

### 3.1 Effect of *Aloe vera* gel and Se NPs on growth parameters

**Figure 1:**
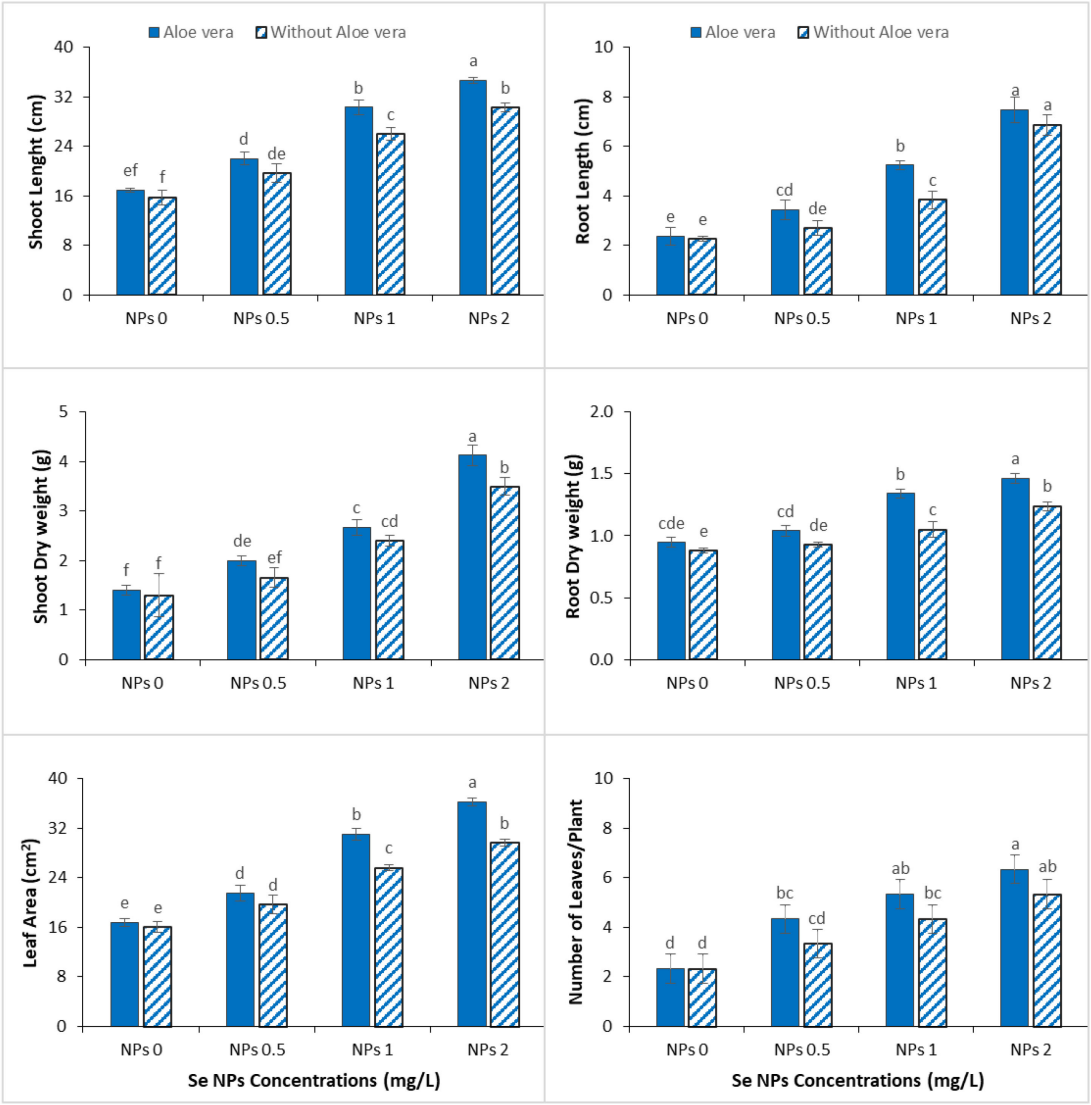
Effect of the co application of *Aloe vera* gel and selenium nanoparticles (Se NPs; 0, 0.5, 1, and 2 ppm) on shoot and root length, shoot and root fresh weight, leaf area, and number of leaves of spinach grown in chromium-contaminated soil. Values are presented as mean ± SD (n = 3). Different letters represent the statistically significant differences among treatments at p < 0.05.

Chromium stress significantly inhibited all growth characteristics of spinach, suggesting its high phytotoxicity on plant development. However, the effect of Se-NPs, alone or in combination with *Aloe vera* gel, was able to significantly reduce this inhibitory effect and enhance plant growth (**Figure 1**). The improvement was dose-dependent, where 2 mg/L Se-NPs had a significantly higher effect than 1 mg/L. At 1 mg/L Se-NPs, significant improvement was recorded in shoot and root elongation, biomass accumulation, leaf area, and leaf numbers and can be considered as partial mitigation of Cr induced stress. This effect was more prominent at 2 mg/L where root growth and biomass demonstrated better development, suggesting that nutrient uptake and overall physiological performance was enhanced under stress conditions. The combined application of *Aloe vera* gel and Se-NPs further enhanced these positive responses in all parameters. Notably the synergistic effect was observed more at a higher concentration of Se-NPs, where maximum improvements in both morphological and biomass related parameters were recorded. This improvement proposes that *Aloe vera* gel may act as a biostimulant, enhancing the efficiency of Se-NPs against Cr toxicity and plant growth.

### 3.2 Impact of *Aloe vera* gel and Se NPs on chlorophyll content

**Figure 2:**
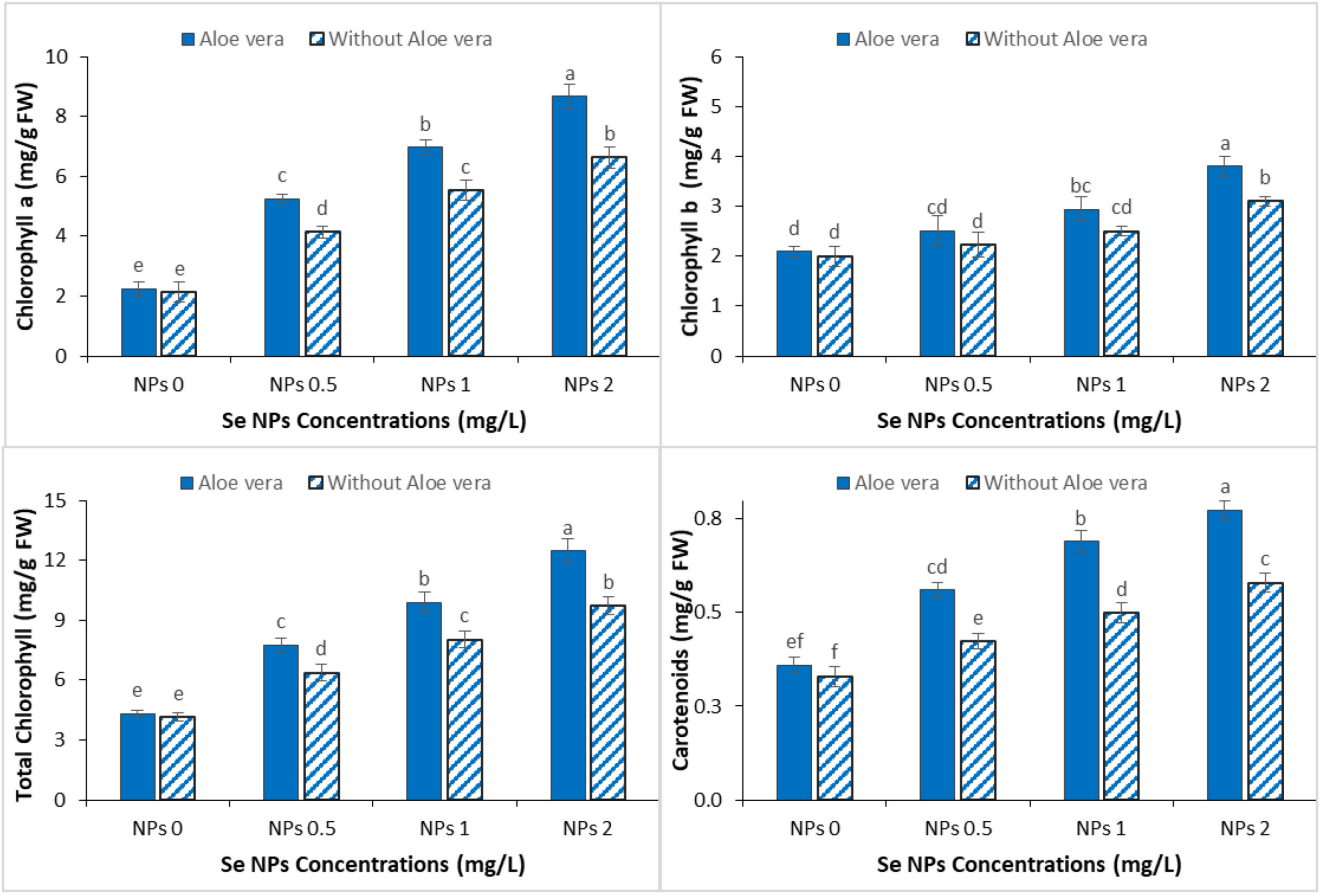
Effect of the combined application of *Aloe vera* gel and selenium nanoparticles (Se NPs; 0, 0.5, 1, and 2 ppm) on chlorophyll a, chlorophyll b, total chlorophyll, and carotenoid contents of spinach grown in chromium-contaminated soil. Values are presented as mean ± SD (n = 3). Different letters indicate statistically significant differences among treatments at p < 0.05.

Chlorophyll pigments in spinach were significantly reduced under Cr stress, reflecting decreased photosynthetic efficiency. However, exogenous application of Se-NPs and *Aloe vera* gel significantly mitigated this reduction and increased the pigment accumulation under Cr stress conditions (**Figure 2**). At 0.5 mg/L Se-NPs, moderate increases were reported for chlorophyll a, chlorophyll b, total chlorophyll and carotenoids indicating partial repair of the photosynthetic apparatus under metal stress. This response became more prominent at 2 mg/L where significant improvements in all pigment fractions were observed suggesting improvement in chloroplast function and increased stability of the photosynthetic system. The combined use of *Aloe vera* gel and Se-NPs further enhanced these beneficial effects in all the measured pigments. Notably, the synergistic effect was more noticeable at the higher concentration of Se-NPs where maximum increases in chlorophyll and carotenoid contents were recorded. This enhancement suggests that *Aloe vera* gel may have bio-stimulant activity to enhance the synthesis of pigments and to shield the photosynthetic machinery from oxidative damage induced by Cr stress.

### 3.3 Effect of *Aloe vera* gel and Se NPs on gas exchange parameters

**Figure 3:**
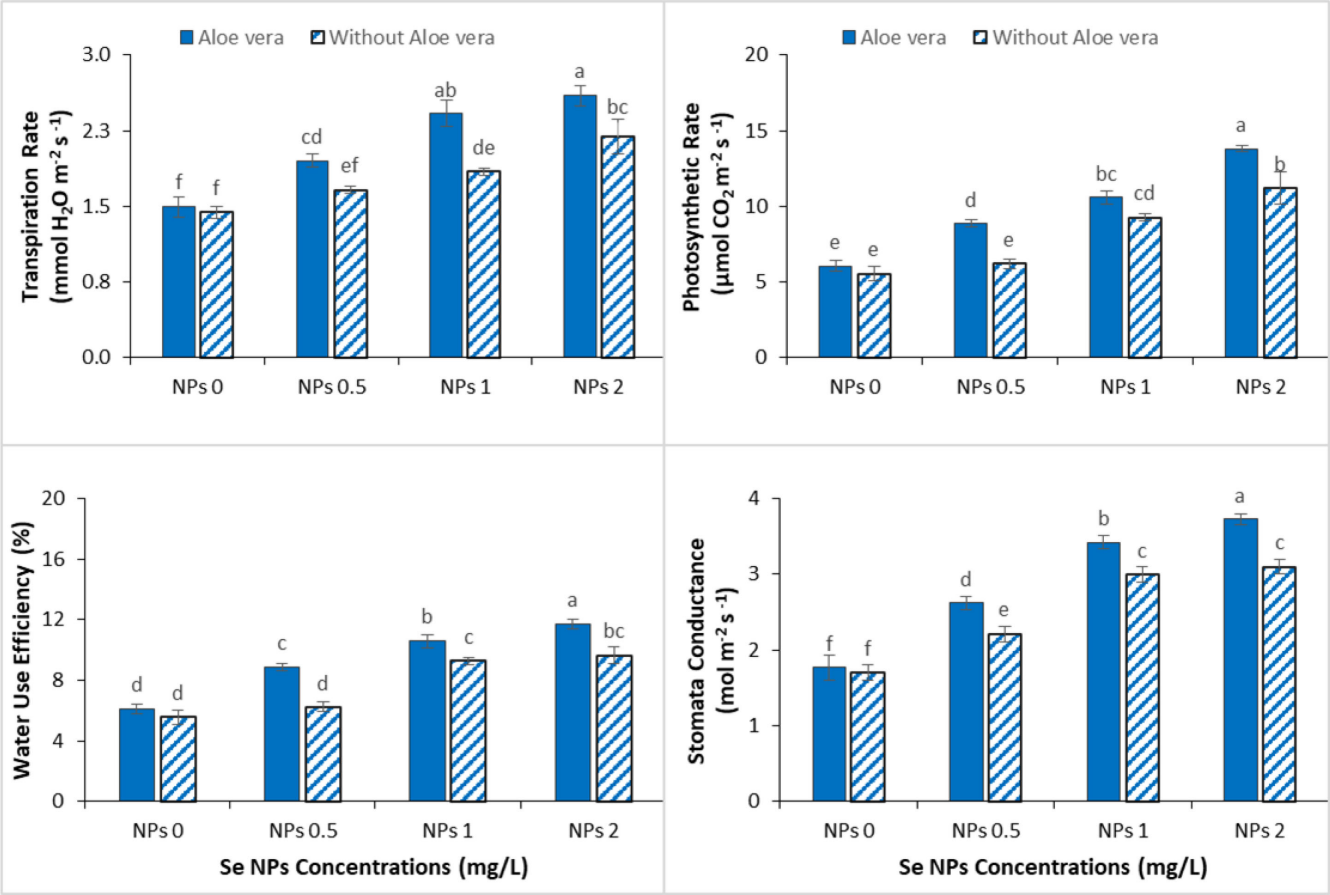
Effect of the combined application of *Aloe vera* gel and selenium nanoparticles (Se NPs; 0, 0.5, 1, and 2 ppm) on transpiration rate, photosynthetic rate, water use efficiency, and stomatal conductance of spinach grown in chromium-contaminated soil. Values are presented as mean ± SD (n = 3). Different letters indicate statistically significant differences among treatments at p < 0.05.

Significant changes in gas exchange parameters of spinach were observed under Cr stress, leading to reduced photosynthetic activity and stomatal regulation (**Figure 3**). However, the exogenous use of Se-NPs, alone or in combination with *Aloe vera* gel, significantly reduced these detrimental effects and enhanced the gas exchange performance under Cr stress. At 2 mg/L Se-NPs, significant enhancements in key gas exchange attributes were found, indicating enhanced stomatal conductance, transpiration rate, and overall photosynthetic efficiency. These results suggest that Se-NPs play a critical role in maintaining physiological processes by reducing the oxidative damage and enhancing cellular function under metal stress. These beneficial effects were further enhanced in the combined application of *Aloe vera* gel with Se-NPs for all the measured parameters. Notably, the synergistic response was observed at both concentrations but was more prominent at 2 mg/L Se-NPs where maximum improvements in gas exchange traits were recorded. This enhancement suggests that *Aloe vera* gel may perform a biostimulant action on the behavior of stomata and thus allow an efficient gas exchange under stress conditions.

### 3.4 Effect of *Aloe vera* gel and Se NPs on oxidative stress parameters

**Figure 4:**
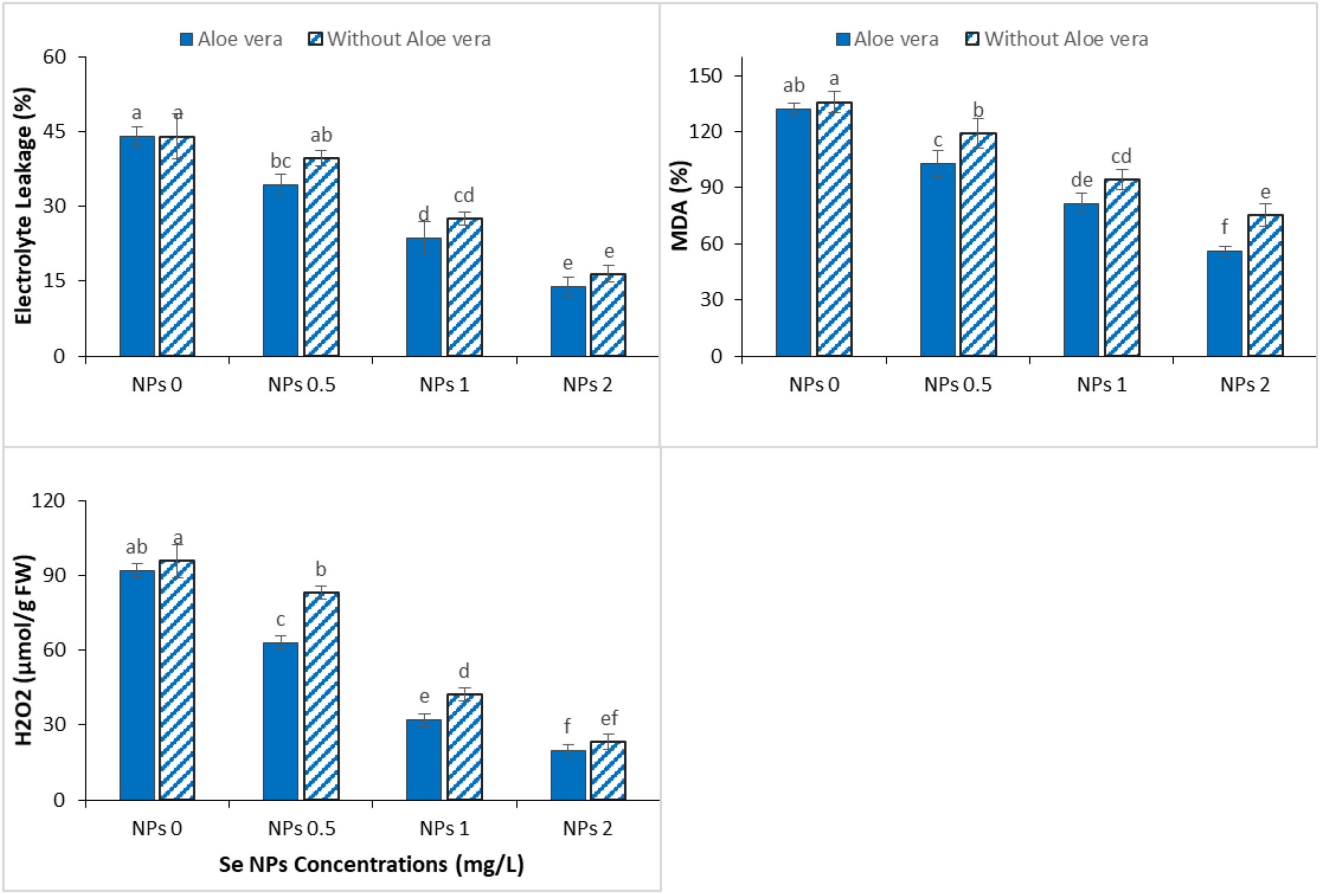
Effect of the combined application of *Aloe vera* gel and selenium nanoparticles (Se NPs; 0, 0.5, 1, and 2 ppm) on malondialdehyde (MDA), electrolyte leakage (EL), and hydrogen peroxide (H_2_O_2_) contents of spinach grown in chromium-contaminated soil. Values are presented as mean ± SD (n = 3). Different letters indicate statistically significant differences among treatments at p < 0.05.

A significant increase in oxidative damage in spinach was observed under Cr stress, as indicated by higher electrolyte leakage, MDA, and H_2_O_2_ levels (**Figure 4**). These changes represent increased membrane instability and oxidative stress in the case of metal toxicity. However, exogenous application of Se-NPs, solely as well as in combination with *Aloe vera* gel, significantly reduced these effects, proving their protective role against Cr-induced oxidative damage. Moderate decreases in electrolyte leakage, MDA and H_2_O_2_ were noted at 1 mg/L Se-NPs, suggesting partial alleviation of oxidative stress. This protective effect was stronger at 2 mg/L, where significant reductions indicated an increase in membrane stability and antioxidant response under stress conditions. The co-application of *Aloe vera* gel and Se-NPs showed an increase in these beneficial effects with maximum reductions in all oxidative stress parameters recorded at 2 mg/L Se-NPs. This synergistic interaction suggests that *Aloe vera* gel increases the effectiveness of Se-NPs in the scavenging of ROS and stabilization of cellular structures.

### 3.5 Impact of *Aloe vera* gel and Se NPs on enzymatic antioxidants attributes

**Figure 5:**
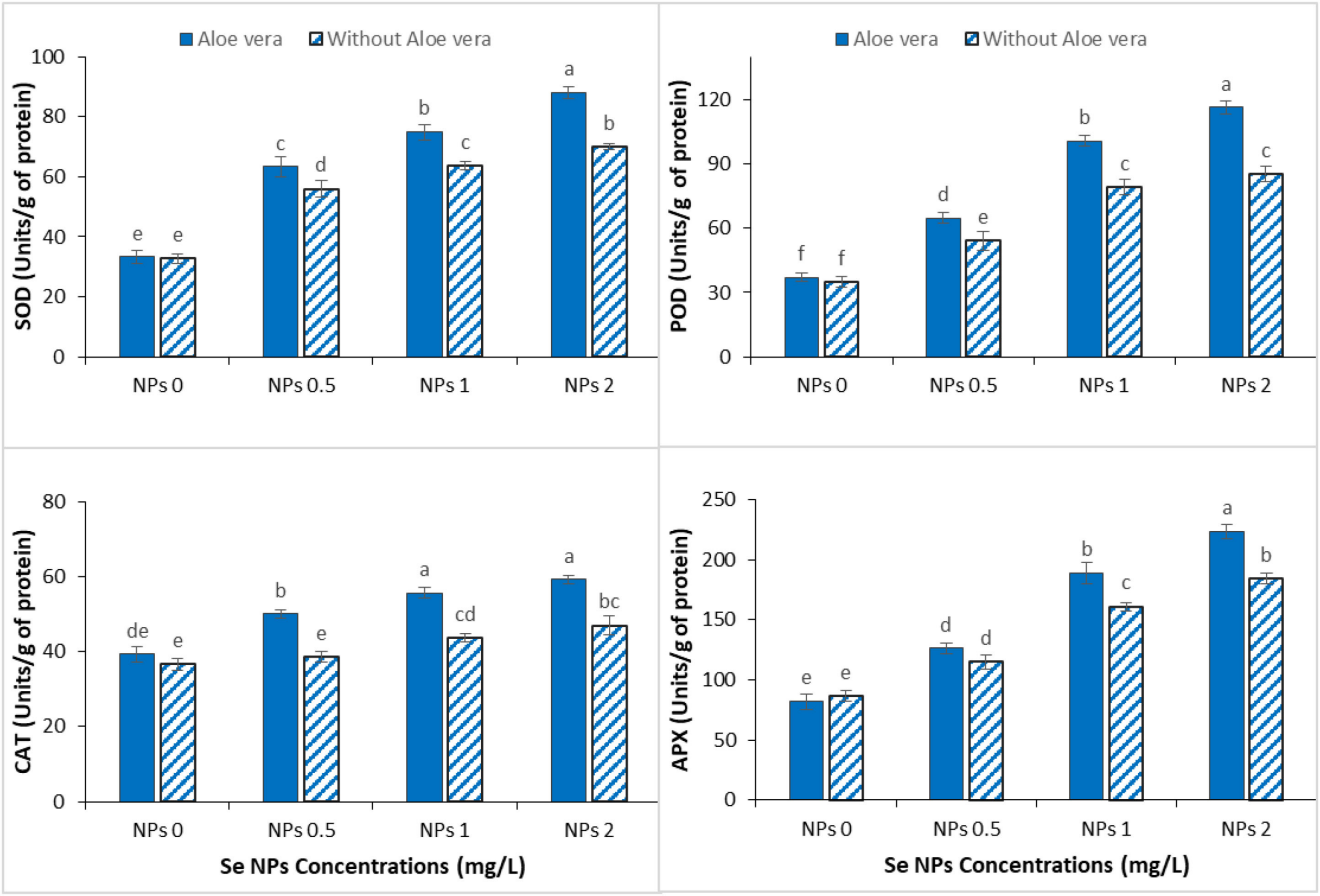
Effect of the combined application of *Aloe vera* gel and selenium nanoparticles (Se NPs; 0, 0.5, 1, and 2 ppm) on superoxide dismutase (SOD), peroxidase (POD), catalase (CAT), and ascorbate peroxidase (APX) activities of spinach grown in chromium-contaminated soil. Values are presented as mean ± SD (n = 3). Different letters indicate statistically significant differences among treatments at p < 0.05.

Cr stress significantly reduced the activity of key enzymatic antioxidants in spinach, such as SOD, POD, CAT and APX, indicating impaired defense against ROS (**Figure 5**). However, exogenous application of Se-NPs alone, or in combination with *Aloe vera* gel, greatly improved these antioxidant defenses under Cr stress, suggesting an improvement in oxidative stress mitigation. Moderate increases in all the measured enzymes were observed at 0.5 mg/L Se-NPs, which can be seen as the partial restoration of the plant’s antioxidative capacity. This response was further enhanced at 2 mg/L Se-NPs, especially for APX and CAT, thus exhibiting a dose-dependent protective effect. The synergistic effect of *Aloe vera* gel with Se-NPs further improved the activities of the enzymes in all treatments. Notably, maximum enhancement in SOD, POD, CAT, and APX was recorded at 2 mg/L Se-NPs with *Aloe vera* gel, suggesting the synergistic effect of Se-NPs with *Aloe vera* gel to enhance the plant to neutralize oxidative stress.

### 3.6 Effect of *Aloe vera* gel and Se NPs on primary metabolites

**Figure 6:**
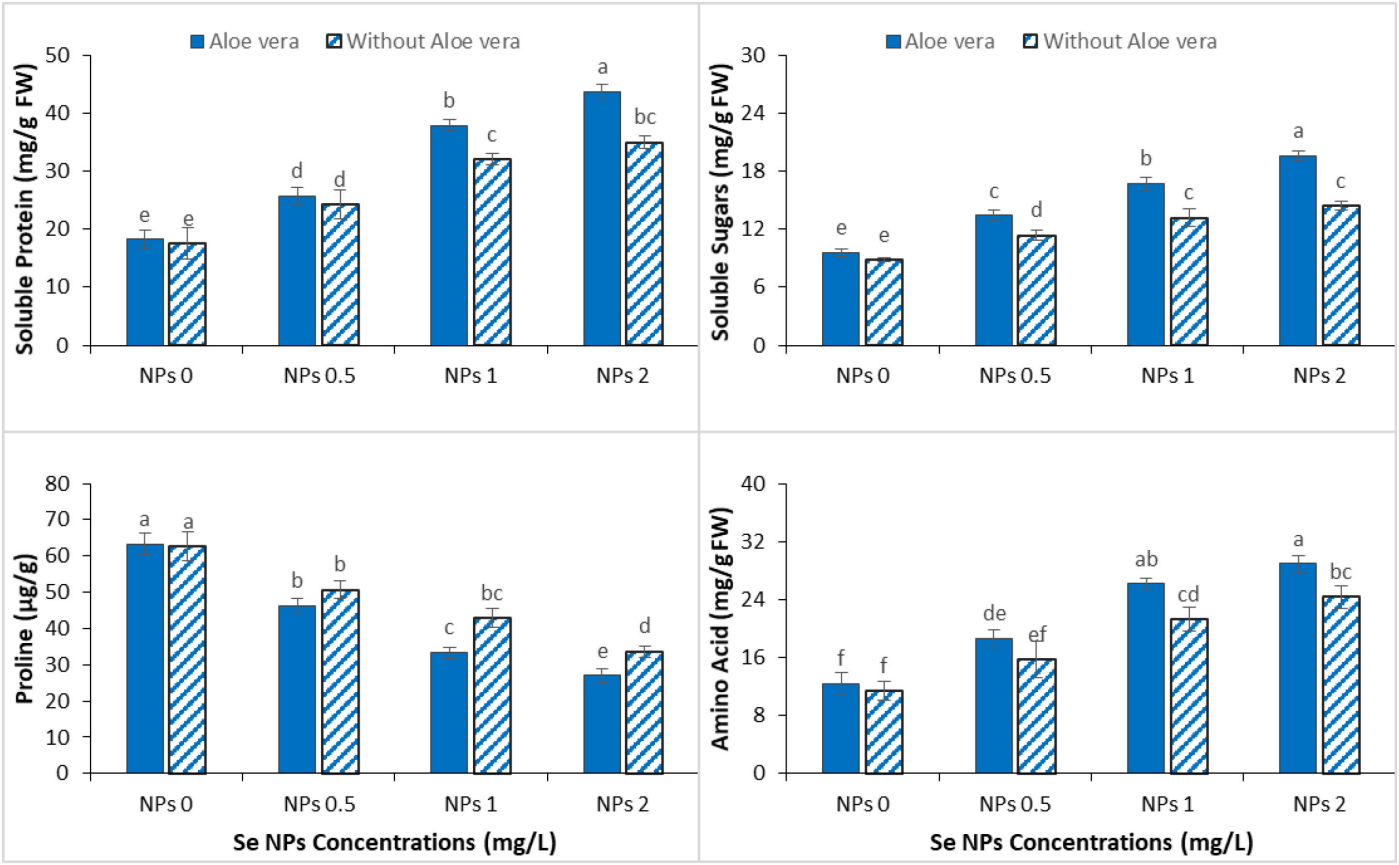
Effect of the combined application of *Aloe vera* gel and selenium nanoparticles (Se NPs; 0, 0.5, 1, and 2 ppm) on soluble protein, soluble sugar, proline, and free amino acid contents of spinach grown in chromium-contaminated soil. Values are presented as mean ± SD (n = 3). Different letters indicate statistically significant differences among treatments at p < 0.05.

Cr stress decreased the concentration of primary metabolites in spinach, such as soluble proteins, soluble sugars, proline and total amino acids, indicating that the basic metabolism was broken and that metabolism was disordered. However, the application of Se-NPs, alone and in combination with *Aloe vera* gel, caused a significant improvement in the levels of these metabolites under Cr stress, suggesting an increased metabolic resilience (**Figure 6**). Moderate increases of all the measured metabolites were observed at 1 mg/L of Se-NPs, which would indicate a partial restoration of physiological functions. This effect increased with 2 mg/L Se-NPs, where significant improvements, especially in amino acids and soluble proteins, indicated improved stress adaptation and osmo-protection. The application of *Aloe vera* gel with Se-NPs further increased these positive responses, where the maximum accumulation of primary metabolites was recorded at 2 mg/L Se-NPs. This synergistic effect suggests that *Aloe vera* gel might boost the metabolic efficiency and stress tolerance of spinach by supporting protein synthesis, osmolyte accumulation and energy metabolism.

### 3.7 Effect of *Aloe vera* gel and Se NPs on Cr

**Figure 7:**
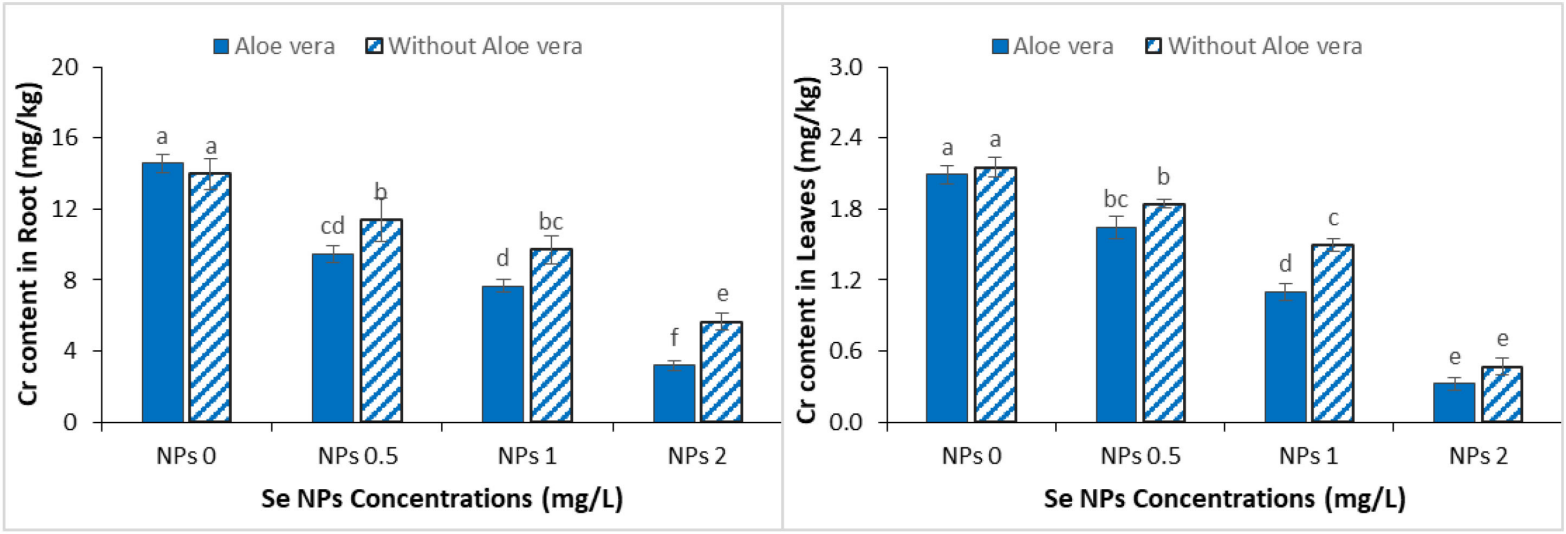
Effect of the combined application of *Aloe vera* gel and selenium nanoparticles (Se NPs; 0, 0.5, 1, and 2 ppm) on chromium (Cr) accumulation in roots and leaves of spinach grown in chromium-contaminated soil. Values are presented as mean ± SD (n = 3). Different letters indicate statistically significant differences among treatments at p < 0.05.

Spinach plants grown in Cr contaminated soil showed high Cr accumulation, demonstrating that Cr is well taken up and translocated. However, exogenous application of Se-NPs, individually and in combination with *Aloe vera* gel significantly reduced Cr uptake, showing an effective mitigation of metal toxicity (**Figure 7**). A moderate reduction in root Cr content was observed at 0.5 mg/L Se-NPs while a significant decrease occurred at 2 mg/L Se-NPs, and a high dose of Se-NPs (2 mg/L) suggesting a dose-dependent effect on protection. The co-application of *Aloe vera* gel further improved this response with the highest decrease in root Cr contents measured at 2 mg/L Se-NPs together with *Aloe vera* gel. These results indicate that *Aloe vera* gel may promote sequestration or immobilization of Cr while Se-NPs may chelate Cr and reduce its translocation, which collectively reduced the accumulation of Cr in spinach roots. In general, the combined treatment was more efficient than Se-NPs alone in reducing Cr uptake and mitigating metal stress.

### 3.8 Explanation of correlation plot

**Figure 8:**
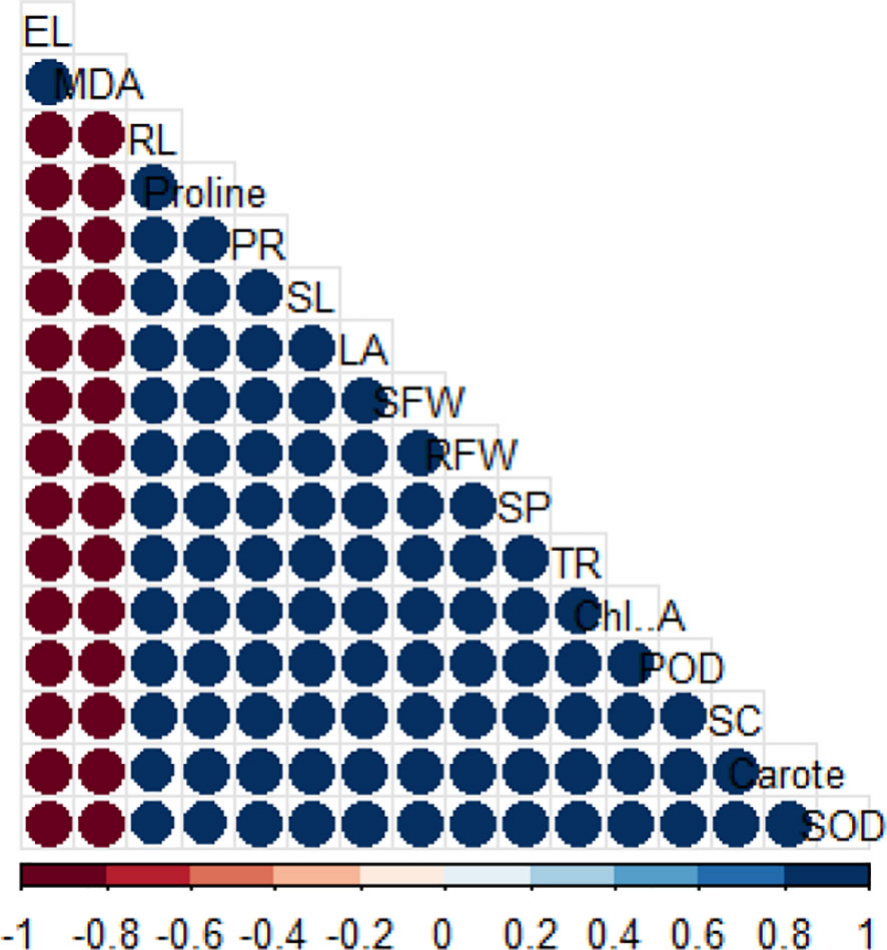
The visualization by a heatmap shows the degree and relationships between multiple physiological and biochemical parameters. The color spectrum ranging from the blue to red quantifies the correlation strength and orientation between parameters where strong positive links appear blue whereas strong negative links appear red. The measurement variables comprise LA (Leaf Area), RL (Root Length), Chl.A (Chlorophyll a), Carotenoids, EL (Electrolyte Leakage), MDA (Malondialdehyde), antioxidant enzymes (SOD, POD), PR (Proline), SL (Shoot Length), SFW (Shoot Fresh Weight), TR (Transpiration Rate), SC (Stomatal Conductance) and RFW (Root Fresh Weight).

A correlation matrix illustrating the relationships among measured variables is shown in **Figure 8**. Positive correlations are shown in blue, while negative correlations are depicted in red, with the intensity of color and size of circles representing the strength of the association. Strong positive correlations were observed among antioxidant enzymes such as SOD, POD, and carotenoids, indicating their coordinated role in oxidative stress tolerance. Growth-related parameters including the leaf area (LA), shoot length (SL), and fresh weight (SFW and RFW) also had positive associations, suggesting that improved growth was linked with enhanced physiological performance. In contrast, stress indicators such as malondialdehyde (MDA) and electrolyte leakage (EL) were negatively correlated with growth and antioxidant parameters, highlighting that higher oxidative damage was inversely related to plant vigor and defense mechanisms. This clear clustering of positive versus negative correlations demonstrates the contrasting roles of protective mechanisms and oxidative injury in plant stress responses.

### 3.9 Explanation of heatmap

**Figure 9:**
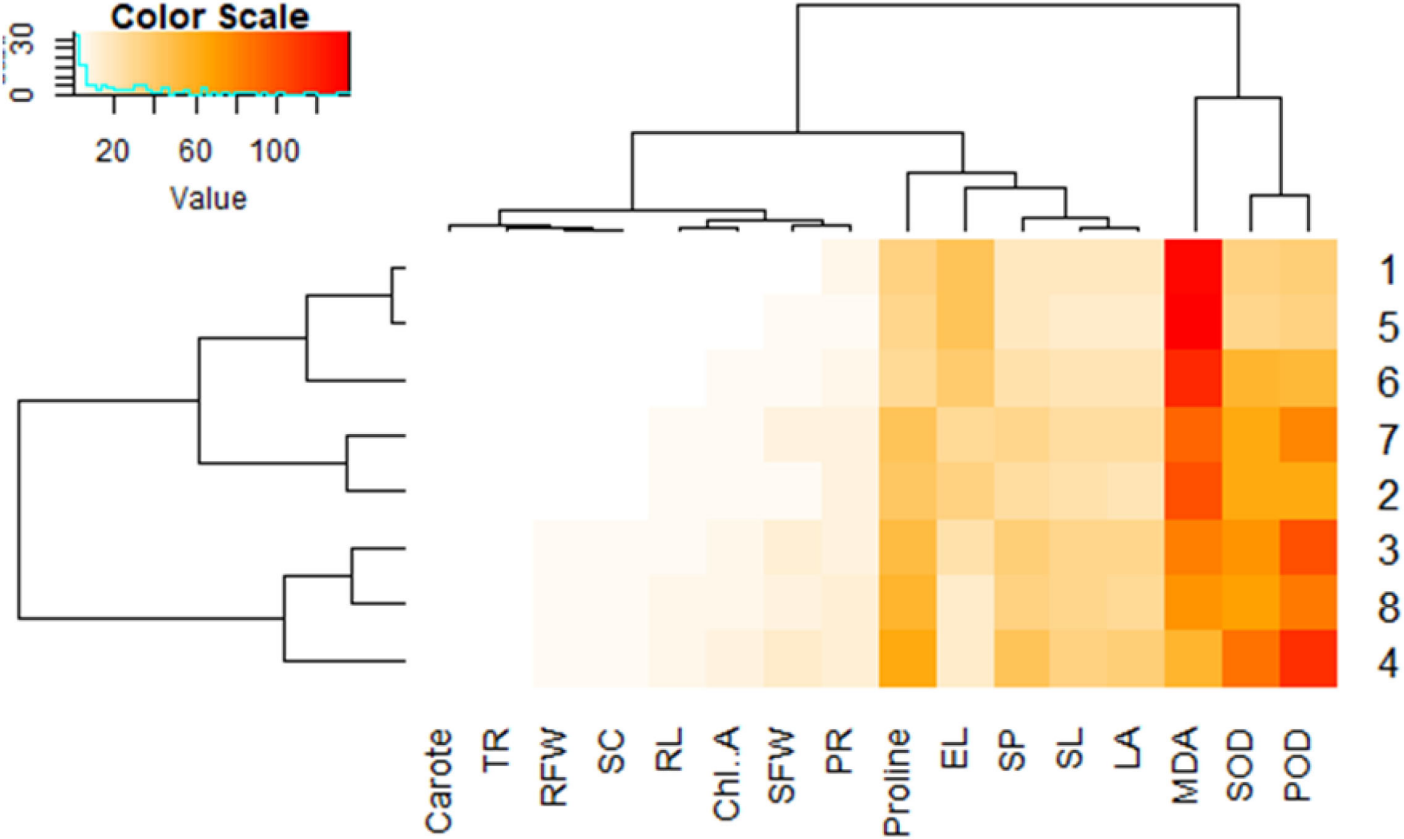
Clustered heatmap showing the expression levels of physiological and biochemical parameters across treatments. High values are indicated in red and low values in pale colors. Dendrograms illustrate the clustering of both traits and samples, revealing patterns of similarity and the relationships among treatments.

The heatmap with hierarchical clustering (**Figure 9**) provides a comparative visualization of variation in biochemical and physiological parameters across treatments. Stress indicators such as MDA and EL clustered together, highlighting their shared role as stress markers. On the other hand, enzymatic antioxidants (SOD and POD) and non-enzymatic traits like carotenoids formed a separate cluster, reflecting a coordinated defense response. Growth related attributes including leaf area, shoot length, and fresh weights were clustered together, reflecting their interrelated contribution to plant biomass production. The color gradient from light yellow to deep red indicates the magnitude of parameter values, with higher intensities for stress markers (e.g., MDA, RL) under severe conditions, while defense-related parameters showed higher values in treated plants. This clustering pattern demonstrates the strong association between the protective effects of bioactive compounds and antioxidants and the maintenance of growth performance under stress.

### 3.10 Explanation of principal component analysis

**Figure 10:**
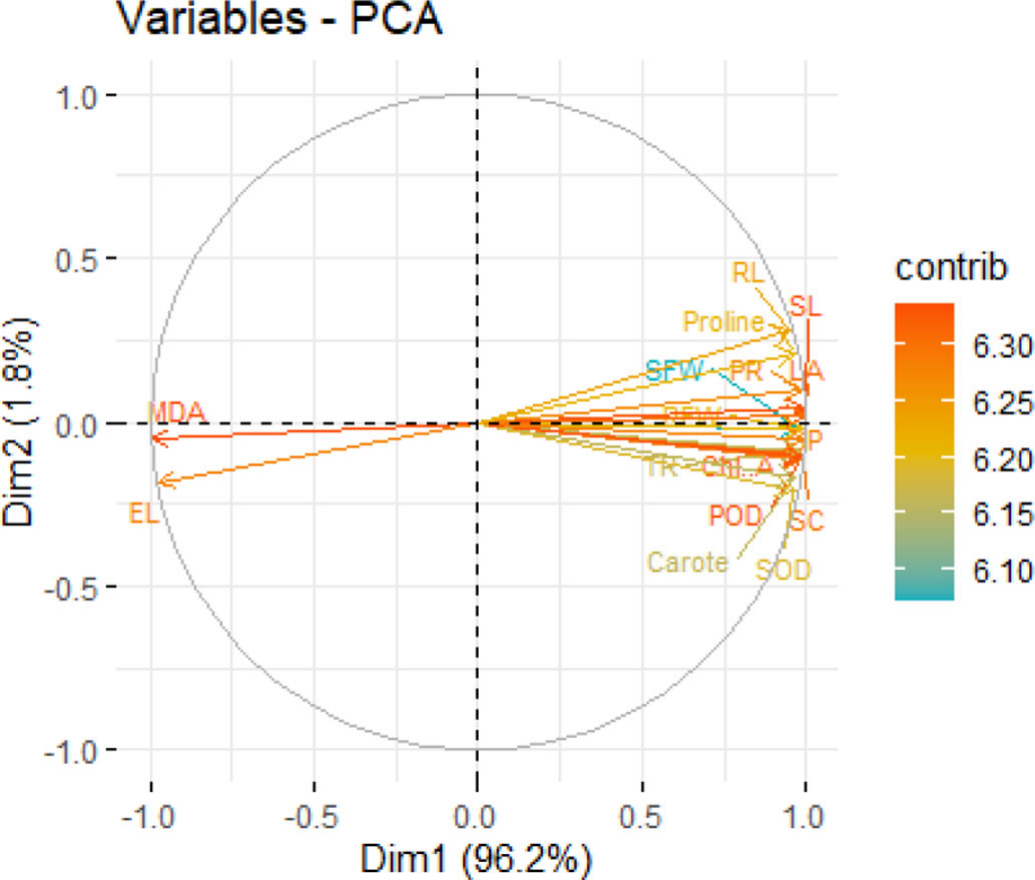
A PCA biplot, based on centered and z-score standardized variables (n = 24 independent pots) shows the relationships among physiological and biochemical parameters associated with chromium stress and several mitigation treatments. In the plot, the length and direction of each of the vectors represent magnitude and sign of the variable loadings, while the color coding indicates the relative contribution of each variable. PC1 and PC2 account for 96.2 and 1.8% of the total variance, respectively.

Principal component analysis (PCA) was performed on variables that had been centered and subjected to z-score standardization and was based on the information in 24 independent experimental units (**Figure 10**). The first principal component (PC1) explained 96.2% of the total variance, showing the existence of a major common gradient of the measured traits. Such a high contribution to variance is statistically reasonable because most of the growth, photosynthetic and antioxidant-related variables showed high positive intercorrelations, whereas the indices of oxidative stress (MDA and EL) were inversely correlated and created a dominant “plant performance - oxidative stress” axis. The second component (PC2) only explained 1.8% of variance and the scree plot showed that there was a sharp decline after the first component, confirming that the remainder of added components contributed little to the overall variation. The loadings supported this structure with high positive contributions of growth and antioxidant traits and negative contributions of markers of oxidative damage.

### 3.11 Chord diagram explanation

**Figure 11:**
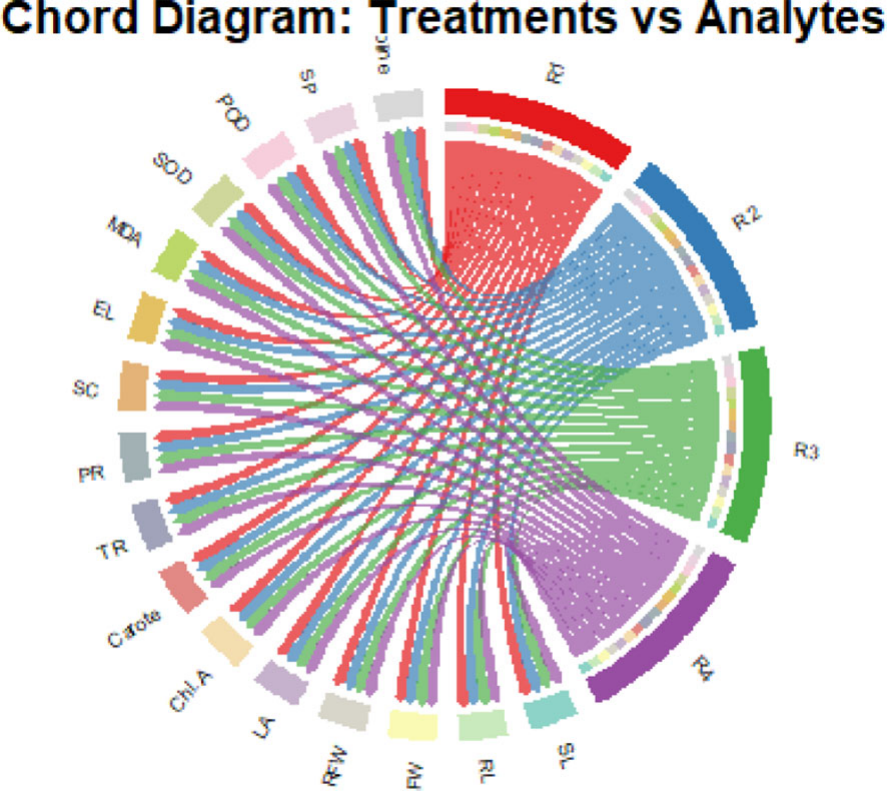
Chord diagram showing correlations between treatments (R1–R4) and measured plant traits. Growth-related traits, including plant height, leaf area, and biomass, are primarily associated with R3 and R4, indicating enhanced growth. Stress-related traits, such as MDA, electrolyte leakage, and antioxidant enzyme activities, are mainly linked to R1 and R2, reflecting higher stress levels. Thicker chords represent stronger correlations, highlighting the combined positive effect of *Aloe vera* gel and Se−NPs in improving growth and antioxidant responses under chromium stress.

Chord diagram in **Figure 11** illustrates the associations between treatments (R1–R4) and physiological and biochemical traits of spinach plants. Treatments are represented as colored arcs (R1–R4) while analysts are shown as opposing arcs. The ribbons connecting the arcs indicate the strength of the association between treatments and traits. R3 and R4 exhibited stronger links with growth-related parameters (SL, RL, SFW, RFW, LA), whereas R1 and R2 were more strongly associated with stress-related markers (MDA, EL, SOD, POD). This visualization highlights the differential effects of treatments on growth enhancement versus stress mitigation mechanisms.

### 3.12 The root-to-shoot translocation factor

The root-to-shoot translocation factor (TF) of chromium in spinach did not exceed from 1 in any NP treatments, ranging from 0.084 to 0.174 (**Table 1**). These values show limited upward movement of Cr and significant retention of Cr in root tissues. A moderate increase in TF was observed at 0.5 mg/L Se-NPs in the presence of *Aloe vera* (0.174), which was suggestive of a slightly increased internal redistribution under low NP exposure. However, the highest concentration of NPs (2 mg/L) showed a significant decrease in TF, especially without *Aloe vera* (0.084), suggesting intensification of sequestration at the root level and reduction of translocation in the shoot under higher stress intensity.

**Table 1:** Root-to-shoot translocation factor of chromium in spinach.

Treatments	NPs (0 mg/L)	NPs (0.5 mg/L)	NPs (1 mg/L)	NPs (2 mg/L)
*Aloe vera*	0.144 ± 0.010	0.174 ± 0.009	0.144 ± 0.014	0.101 ± 0.009
Without *Aloe vera*	0.154 ± 0.011	0.163 ± 0.016	0.155 ± 0.015	0.084 ± 0.019

## 4 Discussion

### 4.1 Effect of *Aloe vera* gel and Se NPs on growth parameters

Cr stress significantly decreased plant growth in the current study as indicated by significant decreases in plant height and biomass. These findings align with prior reports in crops like maize, lettuce and spinach, where Cr toxicity affected the nutrient uptake and plant growth (Malik et al., 2021; Haider et al., 2023; Sami et al., 2023). The observed growth decline in our experiment might be due to decreased photosynthetic efficiency and metabolic disturbances as were reported for *Brassica napus* under Cr stress (Ayyaz et al., 2021). Importantly, the exogenous application of *Aloe vera* gel and Se-NPs significantly reduced these adverse effects resulting in significant improvements in growth parameters as compared with Cr stressed controls. Such improvement is relevant with previous studies, but our results also show a combined effect of both treatments in Cr stress conditions. The improvement of the growth in our research can be explained by the synergistic mechanism of action. *Aloe vera* gel likely has contributed to membrane stabilization and strengthening of the non-enzymatic antioxidant defenses (Shala et al., 2024), while Se-NPs have enhanced the enzymatic antioxidant activities such as SOD, CAT and POD (Dawood et al., 2021). This coordinated response likely restored cellular redox balance, reduced oxidative damage, and maintained normal physiological functioning (Cheng et al., 2023; Wu et al., 2023). Our results are also consistent with the reports on the beneficial effects of Se-NPs on enhancing root growth, nutrient uptake and photosynthic performance of lettuce under HM stress. Contrary to previous studies focused on single treatments, the current research emphasizes the additional advantage of combining Se-NPs with a natural biostimulant to improve mitigation of Cr toxicity. Nevertheless, it is important to mention that beneficial effects of Se-NPs are concentration dependent. While low doses improved growth in our experiment, other papers have reported toxicity at higher concentrations, resulting in oxidative stress and growth inhibition (Burmistrov et al., 2025; Garza-García et al., 2022; Qin et al., 2025). This suggests that careful optimization of the Se-NP dosage is required for practical agricultural applications. Like Cr, other HMs also decrease plant growth, biomass and nutrient uptake, which ultimately affects crop productivity (Hussain et al., 2019). The consistency of these results across different metals provides support for the more general relevance of our results.

### 4.2 Effect of *Aloe vera* gel and Se NPs on chlorophyll content

Cr stress significantly decreased the chlorophyll content and photosynthetic efficiency in spinach, indicating disruption of chlorophyll biosynthesis and impairment of the photosynthetic apparatus. These results are consistent with previous reports showing that Cr toxicity creates extreme morpho-physiological and photosynthetic limitations in plants (Vajpayee et al., 2000; Akhtar et al., 2023; Shafique et al., 2020). The decline observed in our results represents damage in chloroplast structure, electron transport inhibition and reduced CO_2_ assimilation. Notably, the application of *Aloe vera* gel and Se-NPs significantly improved chlorophyll content and photosynthetic performance when compared to the Cr-stressed plants, which suggests a clear recovery of the photosynthetic function. This improvement supports previous results in spinach where such treatments increased nutrient uptake and chlorophyll production (Wahab et al., 2023; Khan et al., 2023), though our results indicate that their synergistic application would result in a greater protective effect. The increased photosynthesis phenomena can be explained by the protective effect of Se-NPs on maintaining chloroplast integrity and assimilation of CO_2_ and by the contribution of *Aloe vera* gel to membrane stabilization and the action on the metabolism. These observations agree with studies that reported Se-NPs increase photosynthetic efficiency and stress tolerance under HM exposure in mungbean (Fatima et al., 2025; Jamshed et al., 2024). However, unlike these studies, which were performed on single applications, the current results describe the presence of a synergistic interaction between *Aloe vera* gel and Se-NPs, which was more effective in terms of chlorophyll level and photosynthetic efficiency maintaining under Cr stress. This suggests that the combined treatment is not only protective to photosynthetic pigments but also improves the antioxidant capacity, thus maintaining redox balance. The enhanced photosynthetic performance that occurred is related to the stabilization of photosynthetic machinery by increased redox control and membrane protection (Liu et al., 2023). Therefore, the concerted treatment seems to act more as a regulator of oxidative status in the cell rather than just an agent of growth. This distinction is important as it can explain the sustained photosynthetic activity and accumulation of biomass under Cr stress conditions in this study.

### 4.3 Effect of *Aloe vera* gel and Se NPs on gas exchange parameters

Cr stress significantly reduced gas exchange parameters in spinach, such as transpiration, stomatal conductance, and photosynthesis, indicating impaired physiological function. These findings are consistent with previous reports in spinach, maize, and rice where Cr toxicity hindered gas exchange by interfering with water relations and metabolic activities (Gupta and Seth, 2021; Razzaq et al., 2023; Alwutayd et al., 2024). The decrease found in our results can be attributed to decreased root functioning and altered ion homeostasis which may have triggered stomatal closure and obstruction of CO_2_ diffusion, as also described by Kumar et al. (2023). However, exogenous application of *Aloe vera* gel and Se-NPs showed an improvement in gas exchange parameters compared to Cr-stressed plants, showing a clear recovery of the physiological performance. This improvement is related to previous studies in tomato and broccoli (Sharma et al., 2024), but our findings further highlight a stronger effect when both treatments were applied in combination. The improved gas exchange in this study indicates improved CO_2_ assimilation and metabolic activity that was likely due to better nutrient availability and activation of key enzymes in photosynthesis. Importantly, the restoration of transpiration and stomatal conductance in our results suggests a restoration of plant water status and stomatal regulation under Cr stress. This could be attributed to improved root hydraulic conductance and membrane stability with increased antioxidant protection facilitated by both *Aloe vera* gel and Se-NPs. Such improvements in water relations are important to maintain carbon assimilation under stress conditions.

### 4.4 Effect of *Aloe vera* gel and Se NPs on oxidative stress parameters

Exposure to Cr stress resulted in enhanced oxidative damage in spinach, as reflected by elevated ROS, H_2_O_2_, and MDA levels, along with root growth inhibition. These results confirm that Cr toxicity causes severe oxidative stress resulting in lipid peroxidation and membrane injury, as reported previously (Elkelish et al., 2024). The elevation in oxidative markers seen in our results is suggestive of the disruption of the redox balance in the cell and loss of membrane integrity under the Cr exposure. However, the combined treatment with *Aloe vera* gel and Se-NPs decreased ROS, H_2_O_2_, and MDA levels, suggesting effective mitigation of oxidative stress. This reduction is consistent with previous studies (Wang et al., 2023), but our results further show that co-application have more powerful protective effect than individual treatment. The improved oxidative status in our study can be attributed to the synergistic role of *Aloe vera* gel, which contributes non-enzymatic antioxidants such as polyphenols and flavonoids, with Se-NPs, which enhance the enzymatic antioxidant systems and directly scavenge ROS (Ahmad et al., 2023). Importantly, the marked decrease of oxidative markers under combined treatment supports an increase of both enzymatic (SOD, CAT, POD) and non-enzymatic defense systems, favoring a better redox homeostasis. This concerted antioxidant response was responsible for membrane stabilization and protection against lipid peroxidation. Furthermore, the recovery of root growth exhibited in our results can be correlated with better membrane integrity and less oxidative injury, which is necessary for root functionality in stress conditions. Therefore, the combined treatment seems to increase the cellular redox protection capacity instead of just decreasing ROS concentration and thus offers a more comprehensive defense against Cr-induced oxidative damage.

### 4.5 Effect of *Aloe vera* gel and Se NPs on enzymatic antioxidants attributes

The Cr stress significantly increased antioxidant enzyme activities in spinach, reflecting activation of defense mechanisms against oxidative damage. This response supports previous results that HM stress stimulates antioxidant enzyme activity as plants try to counter excessive ROS production (Varma and Jangra, 2021). However, despite this natural response, the increased enzyme activity under Cr stress was not sufficient to prevent oxidative damage, as reflected by the increased levels of lipid peroxidation in our results. Notably, the combined application of *Aloe vera* gel and Se-NPs further improved the activities of several important antioxidant enzymes (SOD, POD, CAT, and APX) compared to Cr-stressed plants. This enhancement aligns with previous reports (Kavian et al., 2023; Jamshed et al., 2024), but our results show the co-application leads to a stronger and more coordinated antioxidant response. The increased enzyme activities seen in this study indicate enhanced detoxification of reactive oxygen species, in which superoxide radicals and hydrogen peroxide are efficiently converted to molecules of less harm, thereby decreasing oxidative injury. Importantly, the combined treatment suggests that it does not simply increase stress-induced defense responses but also optimizes the ROS-scavenging network. This concerted regulation of enzymatic antioxidants likely contributed to decreased lipid peroxidation, improved membrane stability and increased Cr tolerance. The role of Se-NPs in direct ROS scavenging, and the contribution of *Aloe vera* gel to the cellular metabolic and antioxidant capacity, offers a mechanistic basis for this combined effect.

### 4.6 Effect of *Aloe vera* gel and Se NPs on primary metabolites

Under Cr stress, nutrient uptake and metabolism in spinach were adversely affected, resulting in decreased soluble proteins and carbohydrates. This decrease can be seen as a symptom of impaired metabolic activity and low availability of essential biomolecules needed for growth and stress tolerance, which is linked with previous findings (Zafar et al., 2024). The decrease in primary metabolites in our results probably contributed to the decrease in growth rate and physiological performance under Cr stress. However, usage of *Aloe vera* gel and Se-NPs significantly restored such metabolic components, with the combination of *Aloe vera* gel and Se-NPs showing the greatest improvement among Cr-stressed plants. This recovery agrees with previous studies found that *Aloe vera* gel increases the accumulation of primary metabolites, whereas Se-NPs increased nutrient uptake, photosynthetic efficiency and root development (Samynathan et al., 2023; Wang et al., 2022). Notably, we found their co-application is more integrative in terms of metabolic benefits than individual treatments. The normalization of soluble proteins and carbohydrates reported during the current study shows the improvement of metabolic homeostasis under Cr stress. This is possibly related to improved balance between carbon and nitrogen metabolism associated with photosynthesis and nutrient assimilation. Such metabolic stabilization has significant importance to continue energy production and to activate stress-responsive pathways.

### 4.7 Effect of *Aloe vera* gel and Se NPs on Cr uptake in of metals concentration

Under Cr-contamination, a significant increase in Cr accumulation was observed in both roots and shoots of spinach, with higher concentrations in roots, indicating the restricted translocation to aerial parts. This pattern supports findings in previous studies showing an increase in Cr in plant tissues resulting in physiological and biochemical damage (Zulfiqar et al., 2022). The high Cr concentrations in our results would likely have contributed to growth inhibition and oxidative stress from earlier. However, the use of Se-NPs significantly reduced the uptake of Cr and its translocation from roots to shoots, with the combination of *Aloe vera* gel with Se-NPs having the most significant effect. This reduction aligns with previous studies (Ragavendran et al., 2024), but our study shows that co-application improves the limitation of the Cr mobility in the plant system. The reduced Cr accumulation in shoots observed in this study can be associated with several mechanisms including immobilization of Cr in root tissues, chelating processes and enhanced antioxidant defense that may \limit metal transport. The uniform low TFs in our results suggested spinach primarily retained Cr in roots and this might suggest a phytostabilization strategy rather than active phytoextraction. In addition, the additional decrease of TFs at the higher Se-NP levels is indicative of increased sequestration through the root system and limited xylem loading, which minimized Cr translocation to aerial parts. Remarkably, the small enhancement in TF at lower NP concentrations in the presence of *Aloe vera* gel may be due to an enhanced physiological activity allowing limited redistribution of Cr without significantly accumulating high concentrations in edible tissues. These findings agree with reports concerning reduction of metal bioavailability by *Aloe vera* gel and also the effectiveness of Se-NPs as barriers against heavy metal uptake (Wu et al., 2024; Ali et al., 2022). In contrast, agents like citric acid have been reported to increase Cr uptake and translocation in sunflower (Mallhi et al., 2020), indicating that the response is dependent on the nature of the amendment used.

## 5 Conclusion

Our study demonstrates that co-application of *Aloe vera* gel and Se−NPs effectively sustains spinach growth under Cr stress, enhancing growth, yield, nutrient uptake, and antioxidant activity while reducing oxidative stress and Cr accumulation. The combined treatment was more effective than either individual application, highlighting synergistic benefits of Cr and *Aloe vera*. NPs offer cost-effectiveness by requiring lower doses and precise delivery, also reducing material and labor inputs. *Aloe vera* gel serves as an organic amendment, improving soil health and plant resilience. Limitations of this study include the controlled experimental conditions and the focus on a single crop. Future research should focus on field scale applications, long-term environmental impacts, and optimizing nano-bio integration for sustainable agriculture.

## Data Availability

The original contributions presented in the study are included in the article/supplementary material. Further inquiries can be directed to the corresponding author.
