# Supplementary material for: Co-application of selenium nanoparticles and Aloe vera gel alleviates chromium toxicity in spinach by modulating antioxidative defense mechanisms
Source: Front Plant Sci. 2026 Apr 29;17:1786570. doi: 10.3389/fpls.2026.1786570 (PMC13191912; doi:10.3389/fpls.2026.1786570)
Supplement: Supplementary file 1 [file DataSheet1.pdf]

**Supplementary Table: ANOVA table for the measured traits**

| Traits | Aloe Vera (A) | NPs Levels (N) | Interaction (A × N) |
|---|---|---|---|
| Shoot Length | 57.33** | 323.29** | 3.91* |
| Root Length | 25.07** | 227.90** | 3.67* |
| Shoot Fresh Weight | 264.02** | 1048.29** | 81.21** |
| Root Fresh Weight | 49.22** | 150.95** | 6.65** |
| Shoot Dry Weight | 15.30** | 154.92** | $1.64^{NS}$ |
| Root Dry Weight | 116.44** | 156.42** | 10.27** |
| Leaf Area | 90.58** | 377.37** | 13.56** |
| Number of Leaves | 10.13** | 40.13** | $1.13^{NS}$ |
| Chlorophyll a | 93.78** | 378.30** | 11.28** |
| Chlorophyll b | 20.35** | 52.97** | $2.43^{NS}$ |
| Total Chlorophyll | 83.08** | 298.40** | 9.85** |
| Carotenoids | 186.47** | 201.85** | 14.33** |
| Transpiration Rate | 69.84** | 102.22** | 7.66** |
| Photosynthetic Rate | 76.96** | 208.33** | 6.65** |
| Water Use Efficiency | 110.47** | 203.54** | 8.49** |
| Stomata Conductance | 78.07** | 318.74** | 7.23** |
| Electrolyte Leakage | 8.20* | 154.49** | $1.22^{NS}$ |
| Malondialdehyde | 31.04** | 161.88** | $2.08^{NS}$ |
| Hydrogen Peroxide | 45.12** | 553.74** | 7.59** |
| Superoxide Dismutase | 106.26** | 483.25** | 16.23** |
| Peroxidase | 162.58** | 537.73** | 24.69** |
| Catalase | 201.75** | 95.92** | 11.72** |
| Ascorbate Peroxidase | 62.86** | 529.56** | 17.03** |
| Soluble Proteins | 35.26** | 191.52** | 7.39** |
| Soluble Sugars | 164.58** | 227.93** | 17.67** |
| Proline | 40.25** | 126.78** | 5.99** |
| Amino Acids | 29.43** | 114.89** | $2.17^{NS}$ |
| Chromium in Root | 27.75** | 215.17** | 6.10** |
| Chromium in Leaf | 48.20** | 659.49** | 5.87** |

** = Significant at $p \le 0.01$; * = Significant at $p \le 0.05$; NS = non-significant
